# Apoptotic Effect of Melittin Purified from Iranian Honey Bee Venom on Human Cervical Cancer HeLa Cell Line

**DOI:** 10.1007/s10989-017-9641-1

**Published:** 2017-11-11

**Authors:** Hannaneh Zarrinnahad, Amir Mahmoodzadeh, Monireh Parviz Hamidi, Mehdi Mahdavi, Ali Moradi, Kamran Pooshang Bagheri, Delavar Shahbazzadeh

**Affiliations:** 1 Biotechnology Research Center, Venom and Biotherapeutic Molecules Lab., Pasteur Institute of Iran, Tehran, Iran; 2 Biochemistry Department, Shiraz University of Medical Sciences, Shiraz, Iran; 3 Immunology Department, Pasteur Institute of Iran, Tehran, Iran; 4 International Campus of Shahid Sadoughi University of Medical Sciences, Yazd, Iran

**Keywords:** Apoptosis, Cervical cancer, HeLa cell line, *Apis Mellifera meda*, Melittin

## Abstract

Melittin, an amphipathic 26-residue peptide, is the main component of honey bee venom. Studies have been demonstrated that melittin has an inhibitory effect on proliferation of cancer cells. However, the precise mechanism of action is not completely understood. In the present study we have shown that purified melittin from Iranian honey bee venom shows anti-cancer effects on human cervical cancer cell line through induction of apoptosis. The venom was collected from Iranian honey bee (*Apis mellifera meda*) and melittin isolated using reversed phase HPLC. Biological activity of melittin was analyzed by hemolytic test on human red blood cells. In order to investigate whether melittin inhibits proliferation of cervical cancer cells, the viability of the melittin treated HeLa cell line was measured via MTT assay. Finally, cell death analysis was performed using Propidum iodide and Annexin V-FITC dual staining. The results showed that the half hemolytic concentration (HD50) induced by mellitin was 0.5 µg/ml in free FBS solution. IC50 obtained after 12 h at 1.8 µg/ml by MTT assay. According to flow cytometric analysis, melittin induced apoptosis at concentrations more than 1 µg/ml. These results suggest that melittin induces apoptotic cell death in cervical cancerous cells as observed by flow cytometric assay. It is concluded that melittin could be regarded as a potential candidate in future studies to discovery of new anticancer agents.

## Introduction

Honey Bee Venom (HBV) is a colorless liquid, with acidic entity (pH 4.5–5.5) (Kim et al. 2007). HBV has at least 28 different active compounds with a distinguished health benefits. Several studies identified biological properties of these compounds and the health benefits of each ingredient (Moon et al. 2008). HBV is composed of various peptides including melittin, apamin, adolapin and Mast cell degranulationpeptide (MCD), enzymes such as phospholipase A2 and hyaloronidase, and different biological amins including histamin and epinephrine (Moon et al. 2008; Zhou et al. 2010). Melittin is the main component of HBV (Komi et al. 2017) and comprise about 50% of the dry weight of venom (Sobral et al. 2016). This cationic peptide has five positive charges without disulfide bond and constitute of 26 amino acid residues (Rady et al. 2017). N-terminal region of melittin has typically hydrophobic amino acids while C-terminal of the peptide is mostly composed of hydrophilic residues (Bramwell et al. 2003). Amphipathic property of melittin permits the peptide to interact with phospholipid membranes and convert it to a water soluble compound (Raghuraman and Chattopadhyay 2004). Melittin has high hemolytic activity in red blood cells too (Uawonggul et al. 2011). Several studies have been demonstrated that melittin has an inhibitory effects on proliferation of various cancer cells via induction of apoptosis, necrosis and lysis (Putz et al. 2006).

Evidences show that the activation of apoptosis process plays a critical role in treatment of cancers. Bee venom and melittin can induce apoptosis in several cancer cell lines such as human lung cancer cell line NCI-H1299(Jang et al. 2003), human ovarian cancer cells SKOV3 and PA-1 (Jo et al. 2012), prostate cancer cell lines LNCaP, DU145, and PC-3 in Rabbit, goat and mouse (Park et al. 2011), human hepatocellular carcinoma cell line Bel-7402 (Li et al. 2006), human leukemic U937 cells and in human breast cancer MCF7 cells (Ip et al. 2008). Furthermore melittin prevents liver cancer cell metastasis through inhibition of the Rac1-Dependent pathway (Rady et al. 2017).

Different malignant cells including small cell lung carcinoma (Jang et al. 2003) and hepatocellular carcinoma (Li et al. 2006) can be destroyed by melittin. It has been reported that melittin induced apoptosis in some cancer cells via activation of caspase-dependent pathway (Jo et al. 2012; Moon et al. 2008; Park et al. 2011). However, the precise mechanism of anti-cancer effects of melittin is not fully known (Moon et al. 2008). Moreover melittin can induce necrosis in several cancerous cells such as gastrointestinal cells (Caco-2 and HT29 cells) (Maher and McClean 2008) and human gastric cancer AGS cells (Mahmoodzadeh et al. 2015).

At the beginning of the third millennium, cervical cancer classified as the second most common cancer between women worldwide and in many low-income countries. There were an estimated 530,000 cases of cervical cancer and 275,000 deaths from the disease in 2008 (Ginsburg et al. 2017).

However today the widespread use of cervical screening programs has dramatically reduced rates of cervical cancer in this countries (Bailey et al. 2016).

In spite of several approaches for the treatment of cervical cancer (Board 2016), it appears that none of them are effective expectedly. Considering the side effects of anti-cancer agents, it is critical to find new effective drugs with fewer side effects against cervical cancer. Different natural substances have been documented to have anti-cancer properties (Shanmugam et al. 2016; Zhou et al. 2016). One of these natural products is bee venom and its main component, melittin (Ghabili et al. 2009).

In the present study, anti-cancer effect of melittin on human cervical cancer cell line was evaluated through induction of apoptosis.

## Materials and Methods

### Reagents, Cell Line

3-(4,5-Dimethyl-2-thiazolyl)-2,5-diphenyl-2H-tetrazolium bromide (MTT), dimethyl sulfoxide (DMSO), triflouroacetic acid (TFA), and acetonitrile (ACN) were purchased from Sigma-Aldrich (St. Louis, MO,USA). Annexin V-FITC apoptosis detection kit wasobtained from Biovision (Mountain View, USA). RPMI-1640, fetal bovine serum (FBS), and Penicillin–Streptomycin (10,000 U/ml) were purchased from Gibco (BRL/Life Technologies).Cervical cancerous cell line (HeLa, NCBI code C115) was obtained from Pasteur Institute of Iran.

### Collection of Honey Bee Venom

A hive of Iranian honey bee (*Apis mellifera meda*) was selected in a bee keeping farm in Koohrang region, Chaharmahale Bakhtiari province, Iran. The venom obtained by bee venom collector through electrical stimulation based on Benton protocol with a slight modification (Benton et al. 1963). A glass aquarium containing wired plates of the venom collector was located on top of the hive. The honey bee was stimulated by electrical shock from 1 to 10 mv with interval of 10 s and the process repeated for 1 min. The bees stung the surface of the glass plate in response to the electrical stimulation. The venom dries rapidly in front of the air. Finally, the dried venom was scraped off by a sharp scalpel, transferred to the laboratory, and stored in − 20 °C until use.

### Preparation of Bee Venom

Bee venom (5 mg) was dissolved in ultra-pure water (100 µl), mixed by a vortex mixer for 2 min and centrifuged at 13,000 × rpm for 10 min at room temperature. The supernatant filtered through a 0.2 µm membrane filter and stored at − 20 °C.

### Purification of Melittin by RP-HPLC

Protein concentration of the prepared venom was measured using Bicinchoninic acid method (Smart BCA protein assay kit, Intronbio-Korea).

Melittin was isolated using a RP-HPLC system (Knauer, Germany) with a C18 column (Knauer, Germany). 0.05% TFA in ultra-pure water and acetonitrile containing 0.05% TFA, designated as solution A and B, were used for eluting the fractions. The column eluted by a linear gradient of solution B from 0 to 60 percent for 55 min at 1 ml/min and the peaks monitored at 214 nm. The collected fractions were lyophilized by a freeze dryer (Christ, 2 alpha-Germany). Lyophilized powder solubilized in 1 ml ultra-pure water and stored at − 20 °C.

In order to verification and Comparison of isolated melittin, traditional melittin from Sigma Company and purified melittin were injected to C18 column at the same conditions.

### Hemolytic Activity

The bioactivity of melittin was evaluated using hemolytic activity on human red blood cells according to Al-Badri et al. protocol with some modification (Sobral et al. 2016). Briefly, heparinized blood from a healthy volunteer was collected, centrifuged at 3500 × rpm for 10 min, and washed with PBS three times. Supernatant was discarded and 2% RBC suspension prepared with PBS. Then melittin was serially diluted in PBS to provide 8, 4, 2, 1, 0.5, 0.25, 0.125, and 0.0625 µg/ml samples in a 96-well plate and 100 µl RBC 2% added to each well. Phosphate buffer saline and Triton X-100(1%) was used as negative and positive control, respectively. The plate was incubated at 37 °C for 2 h and centrifuged at 3000 × rpm for 10 min. 100 µl of the supernatant was transferred from each well into the other 96-well plate and optical density was measured at 540 nm by a micro plate spectrophotometer (Epoch-Biotek—USA). The hemolytic percent was calculated as [(OD_sample_ − OD_neg control_)/(OD_pos control_ − OD_negcontrol_)] × 100. The experiments were carried out in triplicate.

### Cell Culture

The cells were cultured in RPMI-1640 supplemented with 10% FBS, penicillin 100 unit/ml and streptomycin 100 μg/ml, incubated at 37 °C in 5% CO_2_ in humidified condition. The cells were removed with 0.25% Trypsin containing 0.02% EDTA and counted using a hemocytometer using trypan blue staining.

### MTT Assay

In order to determine cytotoxic effect of melittin on HeLa cell line, MTT assay was done. The cells were plated in 96-well plates (Guangzhou Jet Bio-Filtration Products-china) at a density of 40,000 cells/well in 10% FBS supplemented RPMI-1640. The plates were incubated at 37 °C, 5% CO_2_ and 80% humidity for 12 h. The medium removed and three study groups treated with melittin (prepared in FBS free medium) at descending concentrations of 8, 4, 2, 1, 0.5 and 0.25 µg/ml and incubation continued for 6, 12 and 24 h. After each incubation time, 20 µl MTT salt (5 mg/ml) added to each well in the dark condition and incubation continued for 4 h. Then the plates were centrifuged (300 × rpm, 10 min) and the supernatants removed. In the next, DMSO (100 µl) was added to each well to dissolve formazan salt and the absorbance measured at 540 nm by a micro plate spectrophotometer (EPOCH—Biotek, USA). Viability Percent calculated as [(O.D_sample_/O.D_control_)] × 100.

### Morphological Analysis

The cells were seeded at density of 5 × 10^5^ cells/well in 12-well plates containing RPMI-1640 supplemented with 10% FBS and incubated for 12 h. Then the medium was removed, treated with 1, 1.8 and 4 µg/ml melittin (diluted in FBS free medium), and incubation continued for 12 h. The cells were observed by an invert microscope (INV100-FL, BEL-Italy) equipped with phase-contrast lens and finally morphological events compared with untreated cells.

### Apoptosis Analysis

In order to detection of apoptosis in HeLa cells, flowcytometric analysis was performed using Annexin V-FITC Apoptosis Detection Kit according to the manufacturer’s instructions. In summary, the cells were plated in 12-well plate at density of 5 × 10^5^ cells/well and incubated for 4–8 h, followed by adding 1, 1.8 and 4 µg/ml melittin in triplicate for 12 h. After incubation time, the cells were detached with trypsin–EDTA, and resuspended in 500 µl binding buffer. PI (5 µl) and Annexin-V-FITC (5 µl) were added to each well and placed at RT for 5 min. Finally, the samples were analyzed using a flowcytometer (CyFlow SL, Partec-Germany) at 488 nm. Annexin V-FITC and PI stained cells were considered as apoptotic and necrotic cells respectively while viable cells could adsorb none of them.

## Results

### RP-HPLC

**Fig. 1:**
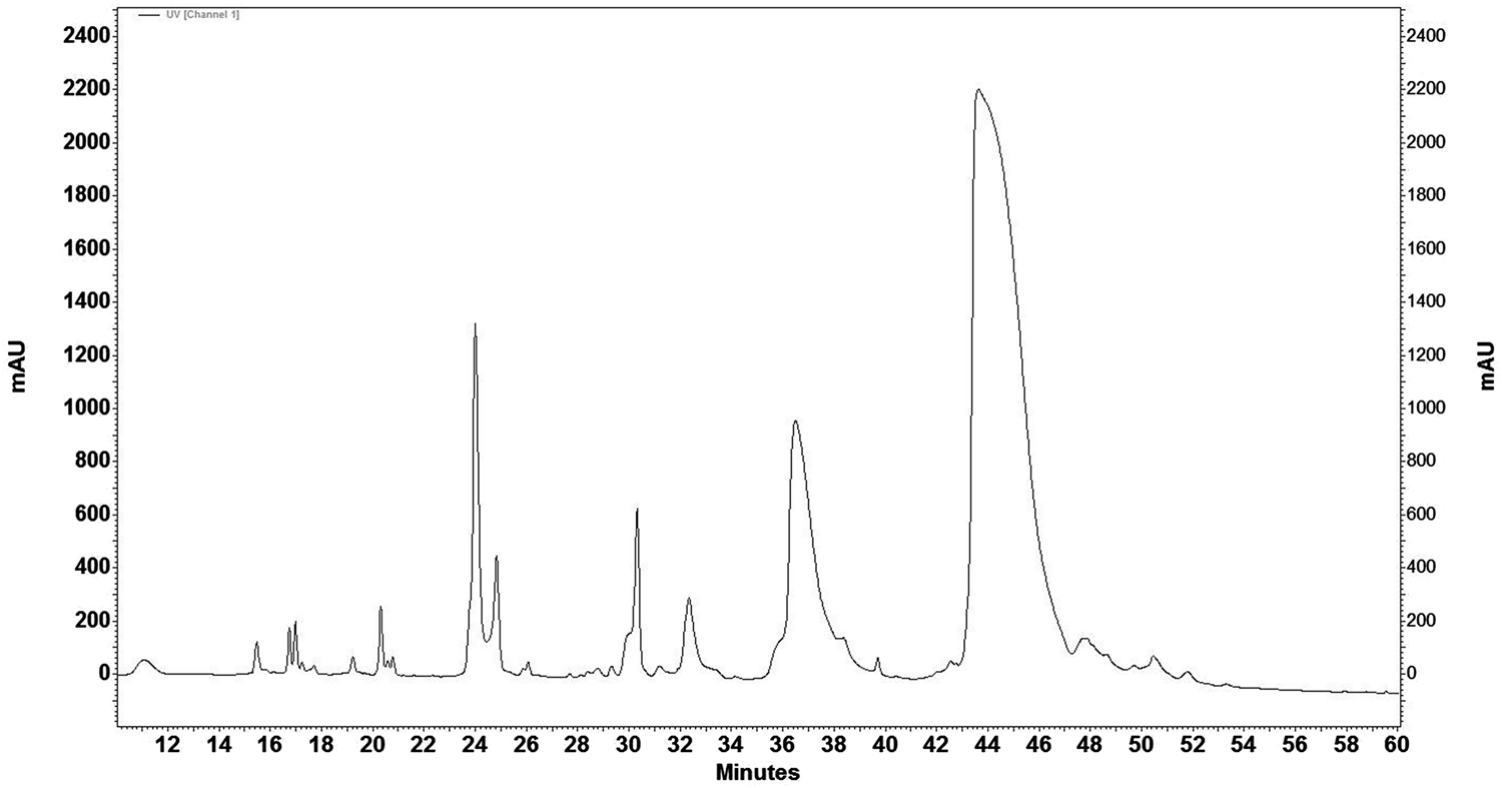
Iranian honey bee venom chromatogram. C18 column (Knauer, Eurosfer-100, 250 × 4.6 mm) was used and a linear gradient method with 0–60% gradient of solution B for 55 min was applied. Flow rate was 1 ml/min in all of the process. The fractions were monitored at 214 nm wavelength. Melittin was eluted at 43 min at 40% of acetonitrile

**Fig. 2:**
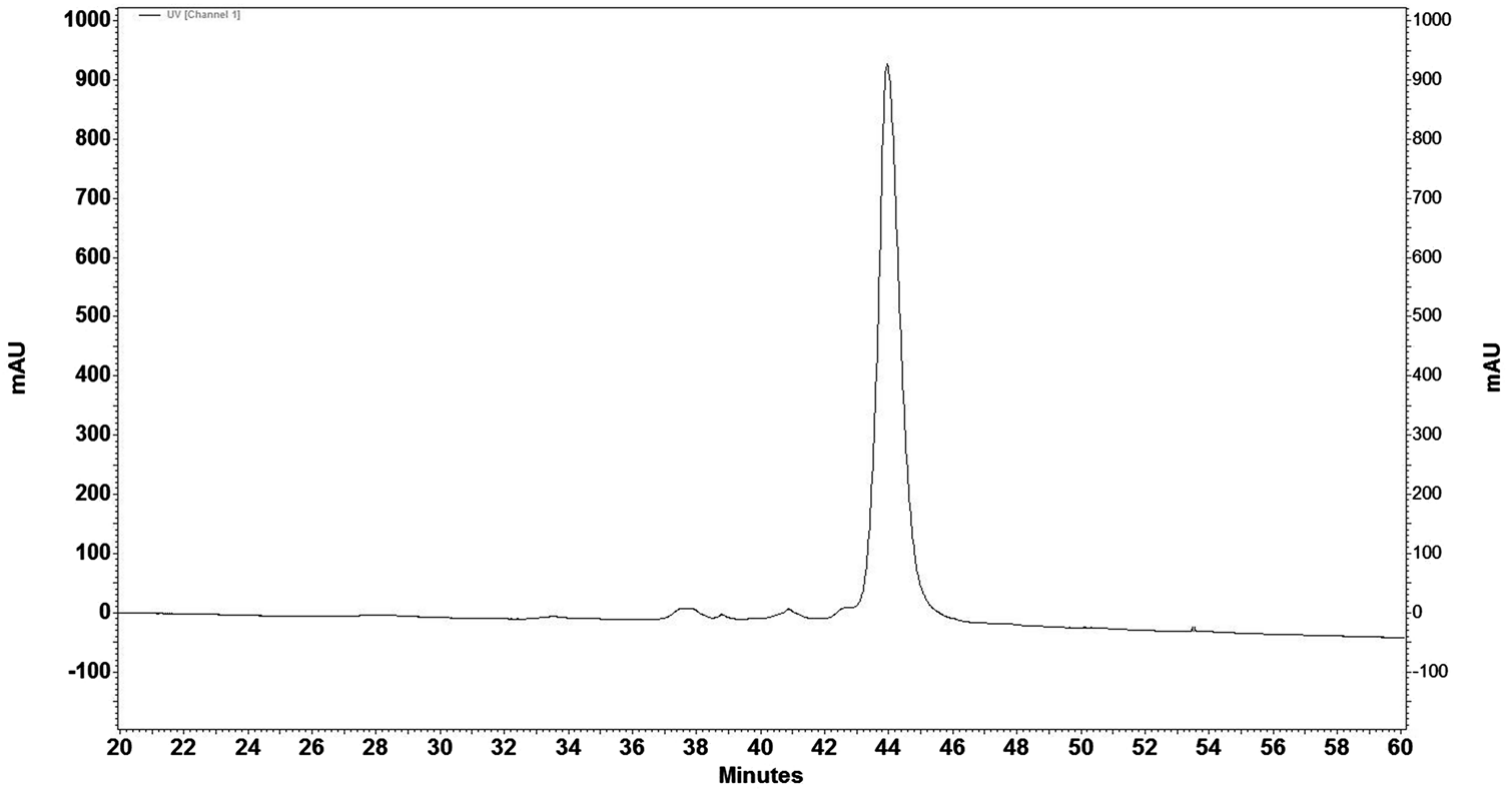
RP-HPLC of purified melittin from Iranian honey BV with the same method was performed. Melittin was eluted at 43.1 min at 40% of acetonitrile. This experiment was done to verify the purity of isolated melittin

**Fig. 3:**
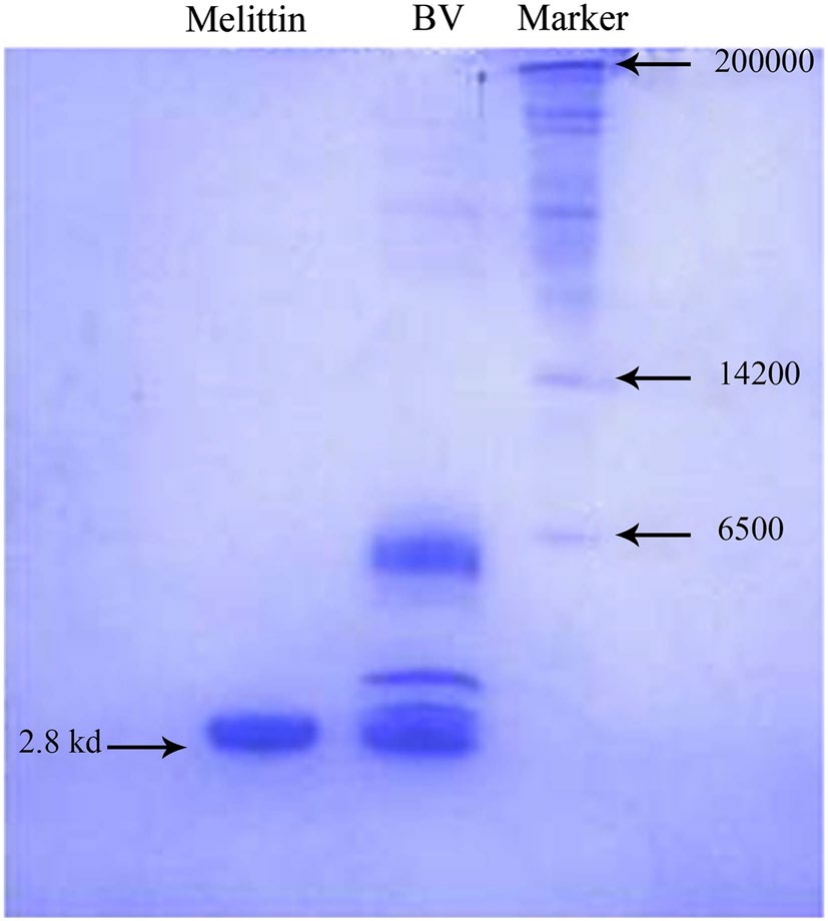
Electrophoresis analysis of isolated melittin from Iranian honey bee venom (SDS-PAGE 15%). Melittin: isolated melittin from Iranian honey bee venom, BV: total bee venom, Marker: protein sixe marker

More than 42 peaks were identified with bee venom using reverse phase HPLC. Among them, 20 major peaks were detected. Melittin was eluted at 43.1 min at 40% acetonitril (Fig. 1). To verify the purity of isolated melittin from Iranian honey BV, it was injected to C18 column with the same method (Fig. 2) in addition SDS-PAGE was performed (Fig. 3).

**Fig. 4:**
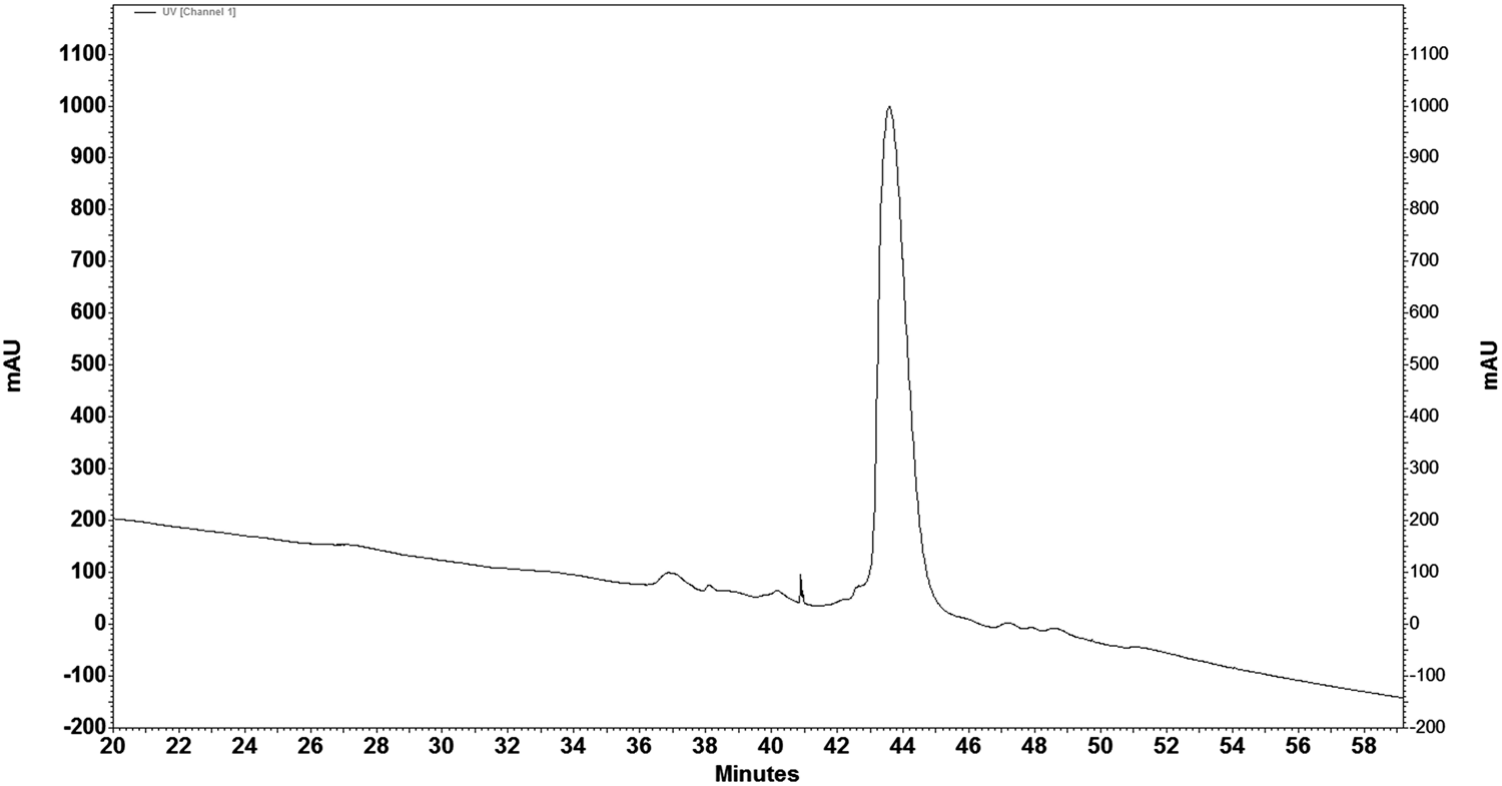
RP-HPLC of mixed melittin from Iranian honey BV and standard Sigma showed the similar retention time at 43.1 ml/min. Chromatogram indicates the same retention time similar pattern for isolated melittin from Iranian bee venom and standard melittin

RP-HPLC of mixed melittin from Iranian honey BV and Sigma standard melittin showed the similar retention time. This experiment demonstrated the purified melittin from Iranian Honey BV contains the same retention time as standard melittin and both of them have the similar pattern (Fig. 4).

### Hemolytic Activity

**Fig. 5:**
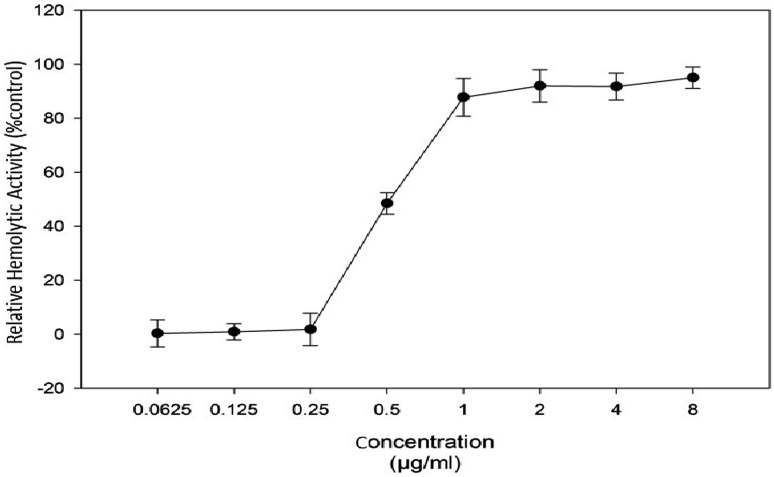
Hemolytic activity assay for melittin at concentration ranged 0.0625–8 µg/ml. The test performed in triplicate and data presented as Mean ± SD

Hemolytic activity assay showed that Melittin had no significant hemolytic activity in lower concentration of 0.25 µg/ml while in concentration more than 1 µg/ml hemolysis was approximately 90% in human RBC in comparison to positive control. HD50 (concentration causing 50% hemolysis of red blood cells) was 0.5 µg/ml (Fig. 5).

### MTT Assay

**Fig. 6:**
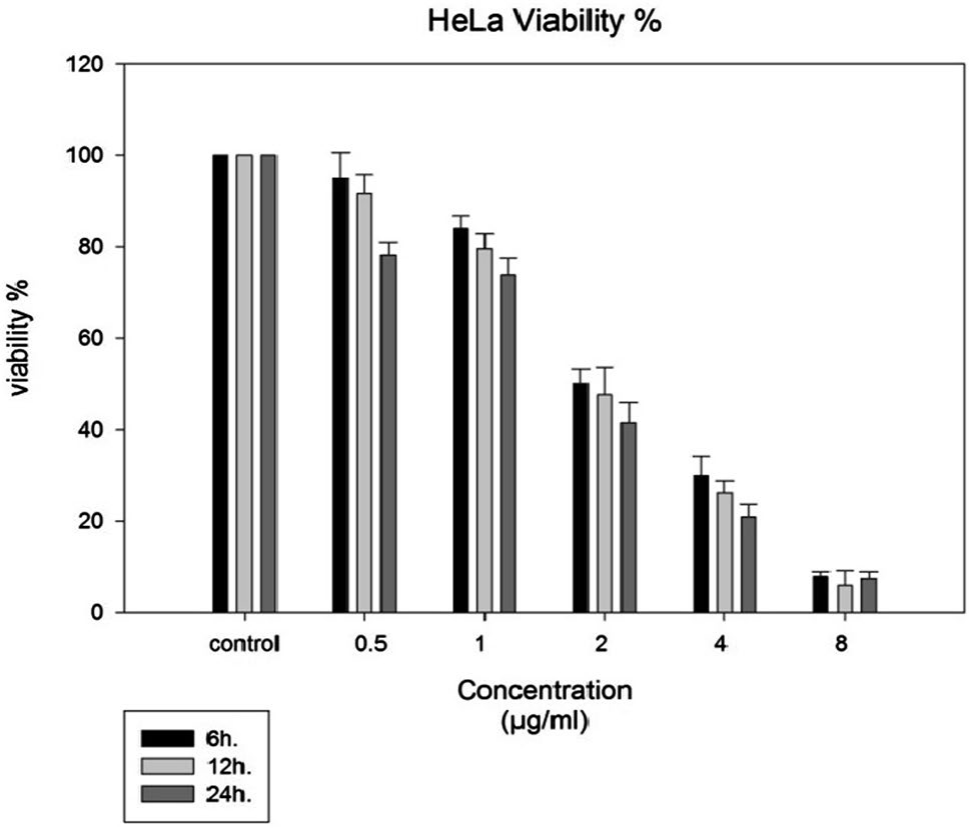
Cytotoxicity effect of melittin on HeLa cell line was determined via MTT assay for 6, 12 and 24 h. The assay was done in triplicate. Results are offered as Mean ± SD. The inhibitory effects of mellittin on HeLa cells are time and dose depended

Results of MTT assay showed that melittin inhibited the proliferation of Hela cell line in a dose and time dependent manner (Fig. 6). IC50 (The half maximal inhibitory concentration of melittin) in 6, 12, and 24 h were 2, 1.8 and 1.7 µg/ml, respectively. Percent of viability for HeLa cells at melittin concentrations 0.5, 1, 2, 4, and 8 µg/ml examined for 6, 12, and 24 h (Table 1). According to the results, the proper time and concentration for the following experiments were selected as 12 h and 1, 1.8 and 4 µg/ml, respectively.

**Table 1:** Percent of viability for HeLa cells at concentrations 0.5, 1, 2, 4, and 8 µg/ml examined for 6, 12, and 24 h

Concentrations µg/ml	0.5	1	2	4	8
Viability % after 6 h	7.85 ± 1.05	30.23 ± 4.18	50.46 ± 3.23	84 ± 2.7	95 ± 5.6
Viability % after 12 h	5.89 ± 3.19	26.17 ± 2.61	47.58 ± 5.98	79.56 ± 3.2	91.67 ± 4
Viability % after 24 h	7.43 ± 1.42	20.88 ± 2.81	41.37 ± 4.46	73.73 ± 3.68	78.1 ± 2.82

### Morphological Evaluations

**Fig. 7:**
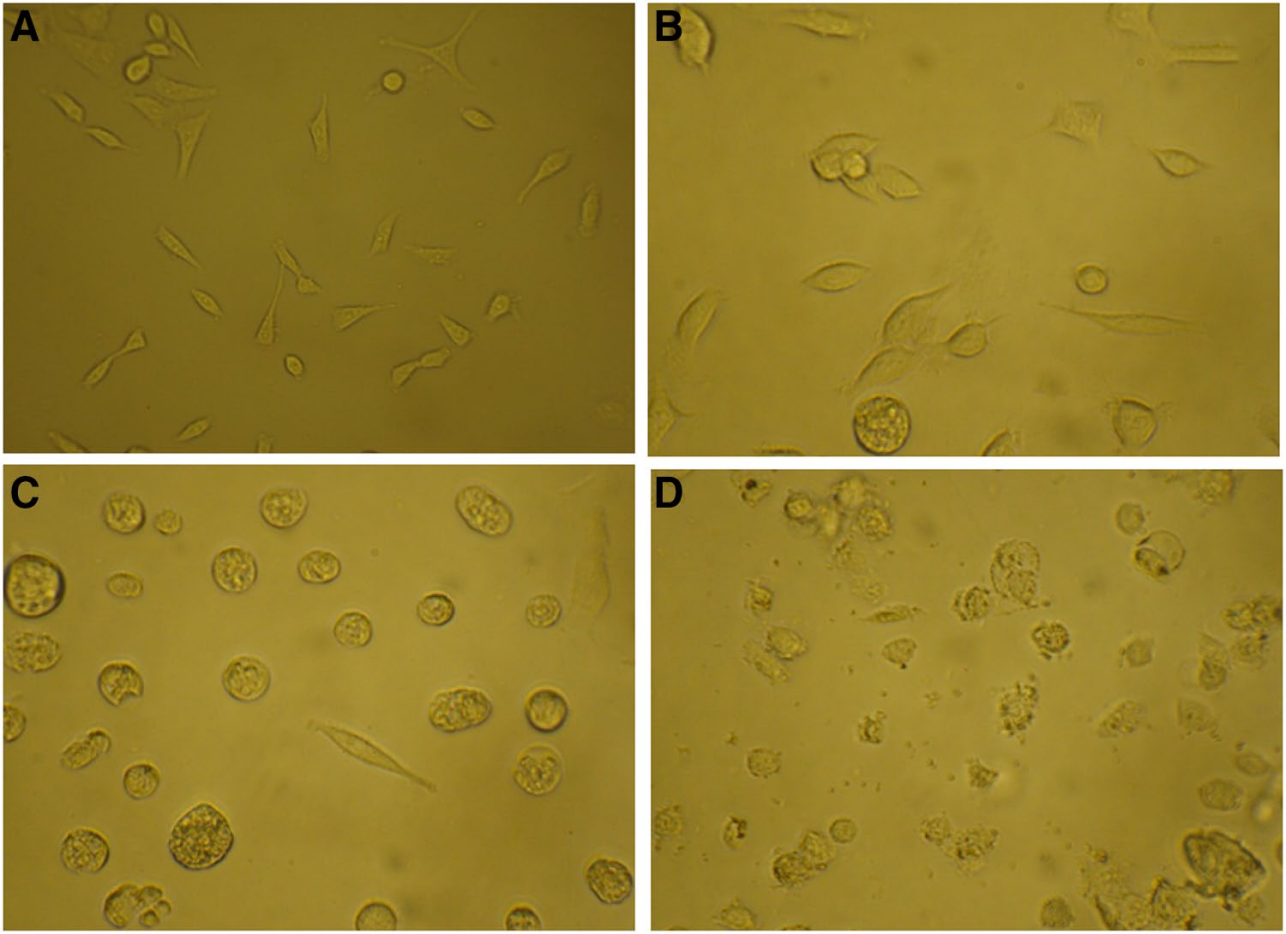
Morphological evaluation of HeLa cells treated with melittin. Negative control (**a**). The cells treated with 1 (**b**), 1.8 (**c**), and 4 μg/ml (**d**) melittin

At IC50 concentration, the major morphological changes included cell shrinkage (Fig. 7b), cytoplasm condensation and disorder in the cell structure (Fig. 7c). HeLa cells crumbled and lost their shape significantly at 4 μg/ml (Fig. 7d).

### Flowcytometry Analysis

**Fig. 8:**
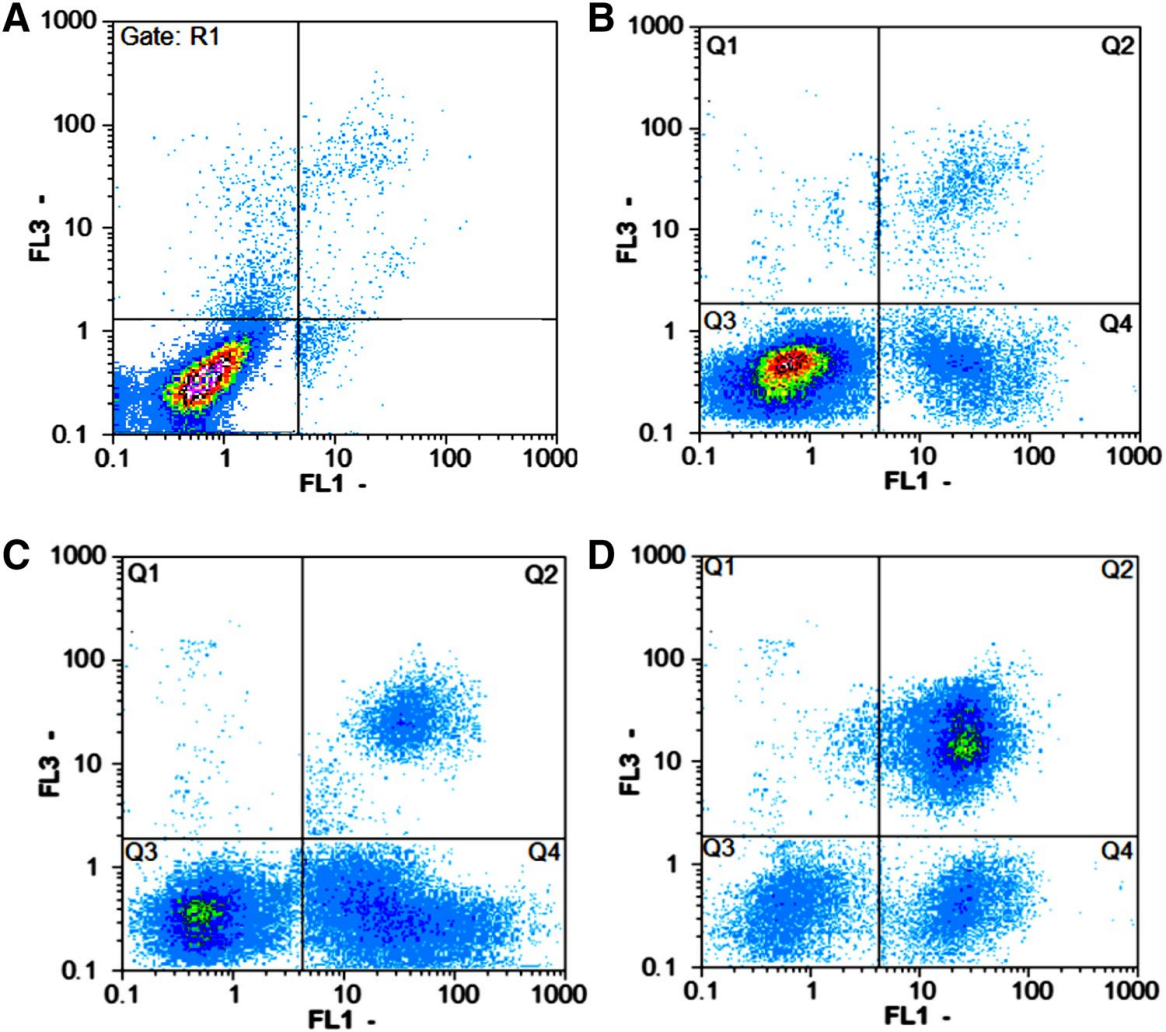
Flowcytometric analysis of HeLa cells treated with melittin concentrations including: **a** control, **b** 1 µg/ml, **c** 1.8 µg/ml, and **d** 4 µg/ml. The quadrants represent the cells at normal state, Q_4_ early and Q_2_ late apoptosis, and Q_1_ necrosis

Flowcytometric analysis of HeLa cells treated with melittin at concentrations of 1, 1.8, and 4 µg/ml is showed in Fig. 8. The quadrants represent the cells at normal state, early and late apoptosis, and necrosis. The distribution of necrotic (PI^+^), late apoptotic (FITC-Annexin V^+^, PI^+^), viable (FITC-Annexin V^−^, PI^−^), and early apoptotic cells (FITC-Annexin V^+^ and PI^−^) incubated with 1, 1.8 and 4 µg/ml melittin are shown in Table 2. According to the results, melittin induce apoptosis in cervical cancerous cells.

**Table 2:** The distribution of necrotic, late apoptotic, living, and apoptotic cells incubated with 1, 1.8, and 4 µg/ml melittin in four quadrants

Concentration µg/ml	Percent of necrotic cells in (Q1)	Percent of late apoptotic cells in (Q2)	Percent of viable cells in (Q3)	Percent of apoptotic cells in (Q4)
0	4.28	1.85	92.22	1.65
1	1.42	10.14	70.22	18.22
1.8	0.9	16.2	47.8	35.1
4	2.6	45.96	22.84	28.7

## Discussion

Natural compounds are one of the significant considerations for treatment of cancers. Among them, honey bee venom and its major component, melittin, have been widely investigated (Jo et al. 2012; Moon et al. 2008). Several studies have been demonstrated that melittin has an inhibitory effect on proliferation of various cancer cell lines including, Human Breast Cancer (Ip et al. 2008), Human Hepatocellular Carcinoma Cell Line (Li et al. 2006), prostate cancer (Park et al. 2011) and ovarian cancer cell lines (Jo et al. 2012) via induction of apoptosis and necrosis (Putz et al. 2006).

Cervical cancer is the seventh most common cancer in worldwide and the third in women (Ginsburg et al. 2017). Surgery, Laser and freezing are generally used to treatment of cervical cancer (Bergmark et al. 2002). In severe cases, chemotherapy and radiation are used to destroy the cancerous cells (Board 2016). In spite of several methods for the treatment of cervical cancer, it appears that none of them are completely effective (Bergmark et al. 2002). Considering the side effects of anti-cancer drugs, it is critical to find new effective drugs with fewer side effects against cervical cancer. Accordingly, this study was aimed to evaluate the effect of melittin on cervical cancer cell line.

Chromatographic analysis in the present study indicated that about 50% of honey bee venom is composed of melittin that is consistent with the report of Helena Rybak (2004). Moreover the number of observed fractions in this chromatogram is similar to previous report (Mahmoodzadeh et al. 2015; Sciani et al. 2010).

Regarding to bioactivity, melittin showed significant hemolytic activity on normal human Red Blood Cells (RBCs) at 0.5 µg/ml. The result is in accordance with the findings in other studies (Gajski et al. 2016; Zhu et al. 2007).

MTT assay showed that melittin inhibits proliferation of HeLa cell line in a dose and time dependent manner that resembled with the results of Jang et al. on NCI-H1299 lung cancer (Jang et al. 2003), Jo et al. in ovarian cancer cells (Jo et al. 2012) and in liver cancer cell metastasis (Liu et al. 2008). IC50 for melittin during 6, 12, and 24 h incubation were 2, 1.8 and 1.7 µg/ml, respectively. The effect of melittin on cell viability has been confirmed in various studies (Jang et al. 2003; Park et al. 2011). The results of MTT in study of Jang et al. (2003) showed a trend of increasing cytotoxicity with increasing concentration and incubation time. Park et al. (2011) demonstrated that bee venom and melittin inhibited cell proliferation in prostate cancer cells in a concentration and time-dependent manner. Liu et al. (2008) have shown the cytotoxic effect of melittin on liver cancer cells. In study of Jang et al. (2003), BV showed dose and time-dependent cytotoxic effects on the NCI-H1299 cell line cells. Park et al. reported the inhibitory effect of BV and melittin on LNCaP, DU145, and PC-3 cells (Park et al. 2011). In study of Liu et al. the IC50 for melittin on MHCC97L cells, MHCC97H cells, Rac1-DA-transfected MHCC97H cells and Rac1-DN-transfected MHCC97H cells were 9.24, 4.06, 3.83 and 25.69 μg/ml respectively (Liu et al. 2008). These studies reported the toxicity of melittin on higher concentration but in the present study, MTT assay showed that melittin kills cancerous cells at IC50 of 1.7, 1.8, and 2 µg/ml during 6, 12, and 24 h respectively. Morphological effects of melittin on HeLa cells observed at IC50 concentration. Morphological alterations increased with increasing in melittin concentration and were in accordance to MTT results.

According to flowcytometric analysis, induction of apoptosis was induced on HeLa cells at concentrations of 1 and 1.8 µg/ml while at 4 µg/ml the cells tended to late apoptosis and necrosis. The results of flowcytometry in present study were similar to results of Chen Wang et al. (Wang et al. 2009) and Miran Jo et al. (2012). Chen Wang et al. demonstrated that melittin induces apoptosis on Hepatocellular Carcinoma Cells (Wang et al. 2009). Miran Jo et al. (2012) investigated that melittin inhibited the growth of SKOV3 and PA-1 ovarian cancer cells by the induction of apoptotic cell death in a dose dependent manner. Melittin, has necrosis effects on some of the cancerous cells too. Mahmoodzadeh et al. (2015) showed that melittin can be induced necrosis on AGS cell lines of gastric cancer.

Melittin is a natural compound that its anti-cancerous properties on different cells was indicated and may be as an appropriate candidate in cancer treatment in the future. Present study showed that isolated melittin from *Apis mellifera meda* venom inhibited the proliferation of HeLa cells and induced apoptosis on them. In conclusion, according to the present study, melittin as a main component of honey bee venom can be used as an anticancer drug with some modifications or various methods such as drug delivery system.
